# DrugPipe: Generative artificial intelligence-assisted virtual screening pipeline for generalizable and efficient drug repurposing

**DOI:** 10.1093/biomethods/bpaf038

**Published:** 2025-05-30

**Authors:** Phuc Pham, Viet Thanh Duy Nguyen, Kyu Hong Cho, Truong-Son Hy

**Affiliations:** AI Center, FPT Software, Ho Chi Minh 715000, Vietnam; Faculty of Computer Science and Engineering, HCMC University of Technology, Ho Chi Minh 715000, Vietnam; AI Center, FPT Software, Ho Chi Minh 715000, Vietnam; Department of Computer Science, University of Alabama at Birmingham, Birmingham, AL 35294, United States; Department of Biology, Indiana State University, Terre Haute, IN 47809, United States; Department of Computer Science, University of Alabama at Birmingham, Birmingham, AL 35294, United States

**Keywords:** drug repurposing, virtual screening, multi-objective optimization, generative AI

## Abstract

Drug repurposing presents a promising strategy to accelerate drug discovery by identifying new therapeutic uses for existing compounds, particularly for diseases with limited or no effective treatment options. We introduce **DrugPipe**, a ‘Generative AI-Assisted Virtual Screening Pipeline’ developed within the target-centric paradigm of drug repurposing, which aims to discover new indications by identifying compounds that interact with a specific protein target. ‘DrugPipe’ integrates generative modeling, binding pocket prediction, and similarity-based retrieval from drug databases to enable a scalable and generalizable *in silico* repurposing workflow. It supports blind virtual screening for any protein target without requiring prior structural or functional annotations, making it especially suited for novel or understudied targets and emerging health threats. By efficiently generating candidate ligands and rapidly retrieving structurally similar approved drugs, ‘DrugPipe’ accelerates the identification and prioritization of repurposable compounds. In comparative evaluations, it achieves hit rate performance comparable to QVina-W, a widely used blind docking tool, while significantly reducing computational time, highlighting its practical value for large-scale virtual screening and data-scarce repurposing scenarios. The full implementation and evaluation details are available at https://github.com/HySonLab/DrugPipe.

## Introduction 

The discovery of new drugs is a costly and time-consuming process, often requiring substantial financial investment and many years of development. Traditional *de novo* drug discovery typically involves identifying a lead compound, followed by extensive preclinical and clinical testing, contributing to high costs and lengthy timelines. These challenges underscore the need for more efficient methods. Recent advancements in drug discovery have been driven by the integration of computational methods and artificial intelligence (AI), which have significantly accelerated the identification and optimization of potential therapeutic compounds, offering new avenues for treating various diseases. AI and computational tools have enhanced the speed and efficiency of drug discovery, reduced costs, improved target specificity, and provided valuable data-driven insights [1, 2]. Additionally, these advancements have supported the development of personalized medicine, enabling therapies tailored to individual genetic and molecular profiles [3]. However, challenges remain. One major issue is that while generative AI models can create ligands with high theoretical binding affinities and desirable pharmacological properties, these ligands may be difficult or even impossible to synthesize in practice. This challenge arises because generative AI models often prioritize certain chemical and biological criteria that do not always align with practical constraints in chemical synthesis and manufacturing, leading many promising AI-generated ligands to fail when transitioning from computational predictions to tangible, testable compounds.

Conversely, drug repurposing offers a promising alternative by discovering new therapeutic applications for existing drugs [4]. A key advantage of repurposing is the potential for substantial time and cost savings, as it involves compounds that have already been tested extensively for safety and efficacy, allowing for quicker transitions from research to clinical applications. This is especially crucial in urgent scenarios, such as pandemics, where the lengthy process of developing new drugs from scratch is impractical. Repurposing offers a faster response by leveraging the known safety and efficacy profiles of existing drugs to address emerging health threats. Furthermore, drug repurposing carries lower risk because it relies on drugs with well-documented pharmacokinetic and pharmacodynamic profiles, reducing the likelihood of unexpected adverse effects and increasing the chances of regulatory approval. However, drug repurposing faces significant challenges, such as the extensive time and resources needed to screen a vast number of existing drugs against new targets, making the process resource-intensive and time-consuming [5]. Additionally, many repurposing efforts have been limited to single diseases, restricting the broader applicability of findings and potentially missing opportunities to repurpose drugs for other conditions [6–9].

To address these challenges, we propose an ‘AI-assisted Virtual Screening Pipeline,’ named ‘DrugPipe,’ which offers an efficient and generalizable framework for comprehensive *in silico* drug repurposing. Aligned with the target-centric paradigm of drug repositioning [10], ‘DrugPipe’ begins with a protein target, often novel or understudied, and identifies potential therapeutic compounds based on structural similarity to AI-generated ligand candidates. The pipeline integrates generative modeling for ligand design, binding pocket prediction, and similarity-based retrieval, enabling scalable and efficient screening across large drug libraries. By leveraging the speed and scalability of similarity search—which is substantially faster than conventional docking simulations [11]—‘DrugPipe’ supports rapid, target-specific compound prioritization, even in scenarios with limited prior knowledge. While docking simulations offer detailed insights into molecular interactions, their high computational cost limits scalability. ‘DrugPipe’ addresses this by performing enrichment prior to docking, effectively narrowing the candidate space and allowing computational resources to be concentrated on the most promising compounds. Its modular and extensible design makes it applicable across a wide range of therapeutic areas, including emerging and poorly characterized targets. By combining structure-driven screening with generative modeling, ‘DrugPipe’ delivers a practical and scalable solution for end-to-end computational drug repurposing, enabling both the identification and prioritization of repurposable drug candidates.

To evaluate the generalizability and practical utility of our pipeline, we conducted a comprehensive study using the DrugBank database, which includes protein targets and their corresponding approved drugs. This evaluation aimed to determine whether our pipeline could effectively identify real drugs in repurposing scenarios, such as COVID-19 and HIV. In addition to assessing the overall performance, we compared ‘DrugPipe’ with a standard virtual screening pipeline based on blind docking using QVina-W. This comparison allowed us to benchmark the efficiency and accuracy of our generative AI-assisted approach against traditional structure-based methods. Furthermore, we investigated whether AI-generated ligands could serve as effective substitutes for known drugs in the similarity search process—an especially valuable capability for novel or under-explored targets where no approved drugs are available. By using generated ligands as reference queries, we examined their ability to retrieve approved drugs from large-scale databases based on structural and functional similarity, thus evaluating their potential to initiate repurposing in data-scarce settings. As our model prioritizes compounds by predicted binding affinity, we hypothesize that the top-ranked candidates will include therapeutically relevant drugs. However, acknowledging that approved drugs may not always exhibit the highest binding affinities due to considerations like pharmacokinetics and safety, we also report the distribution of the rank positions at which the first approved drug appears for each target. This analysis demonstrates the pipeline’s ability to reduce the experimental search space and prioritize real, approved drugs, highlighting both the effectiveness and broad applicability of our framework.

The contributions of this work are summarized as follows:

- We present an AI-Assisted Virtual Screening Pipeline (DrugPipe) that aims to support efficient and generalizable *in silico* drug repurposing, particularly in low-data scenarios where no known ligands or drug–target interaction data are available.
- Our study investigates the use of AI-generated ligands as surrogates for known drugs in similarity-based virtual screening, offering practical insights into the challenges and opportunities of applying generative models in real-world repurposing tasks.
- While current generative approaches have limitations, our modular pipeline provides a flexible foundation that can integrate future advances in generative modeling, molecular representation, and screening strategies, helping guide further research in this area.

## Related work

### Generative AI models in drug discovery

Generative AI models have become powerful tools in drug discovery, enabling the design and optimization of novel molecular structures with specific desired properties [12–14]. Numerous studies have demonstrated the effectiveness of these models in generating promising drug candidates. For instance, many generative approaches have produced molecules by conditioning on specific protein binding pockets, resulting in high theoretical binding affinities and pharmacological properties [14–16]. These studies have shown substantial success in the design of ligands tailored to known protein targets. However, a significant limitation of these methods is their reliance on detailed structural information about the protein binding pocket. This dependency becomes a critical drawback in situations where such information is unavailable, as in the case of emerging pathogens like new viruses.

To address this limitation, in this work, we introduce an approach that integrates binding pocket prediction with generative AI models to enhance drug discovery in contexts where binding pocket details are not available. Our method first employs advanced binding pocket prediction techniques to identify multiple potential binding sites on target proteins. Subsequently, a generative AI model is used to design novel compounds optimized for each of these predicted pockets. This strategy not only enables the discovery of new drug candidates when binding pocket information is limited but also enhances the diversity of generated compounds by targeting a variety of potential binding sites, thereby increasing the chances of identifying effective therapeutic agents.

### Current approaches in virtual screening for drug repurposing

Virtual screening has become an essential tool in the drug repurposing process, particularly for the rapid identification of therapeutic candidates for emerging diseases. These approaches utilize computational methods to efficiently screen extensive compound libraries, providing significant advantages in terms of speed and cost–effectiveness compared to traditional drug discovery processes [17].

Despite promising outcomes, several limitations persist across existing research efforts. As summarized in Table 1, many virtual screening and drug repurposing strategies depend on extensive prior knowledge about the protein target, such as detailed information on binding pockets, approved drugs, and known active compounds. This reliance can be a significant drawback, especially when dealing with novel or poorly characterized targets, where such data may be incomplete or unavailable. Additionally, a substantial portion of existing studies relies heavily on docking simulations using tools like AutoDock Vina [6, 19–21]. Although Vina is well-regarded for its accuracy, the process of docking compounds can vary significantly in duration. For simpler receptors and suitable ligands, docking might be completed in minutes. However, with complex protein structures or incompatible ligands, simulations can take several hours per compound. This extended time is due to the need for the algorithm to explore numerous potential binding conformations and interactions, especially when the ligand does not easily fit the binding site. Such variability makes large-scale docking simulations resource-intensive and can significantly slow down the drug discovery process when screening extensive compound libraries [22].

**Table 1.** Comparison of virtual screening for drug repurposing approaches based on input and output characteristics.

Approach	Input			Output	
	Protein structure	Known active compounds	Binding pocket	Drug candidates	Binding poses
Jang et al. [6]	✓	✓	✓	✓	✓
Morselli et al. [18]	✓	✓	×	✓	×
Chen et al. [19]	✓	×	✓	✓	✓
Gan et al. [20]	✓	×	×	✓	✓
Pirolli et al. [21]	✓	×	×	✓	✓
Ours	✓	×	×	✓	✓

Many existing approaches are tailored to specific diseases [6, 18, 21, 23], limiting their generalizability and broader applicability. This narrow focus can hinder the discovery of repurposing opportunities across different therapeutic areas, reducing their overall impact. Moreover, such specificity poses significant challenges when addressing emerging health threats, as these approaches may struggle to adapt to novel pathogens. Rapid and flexible response capabilities are essential for addressing new diseases, where prior detailed knowledge of targets is often unavailable.

Given these limitations, there is a clear need for more versatile and efficient approaches in virtual screening and drug repurposing. Our work addresses these challenges by developing a generalizable framework that minimizes reliance on detailed prior knowledge of protein targets. By incorporating generative AI models for ligand design, the framework eliminates the need for known active compounds and reduces dependency on resource-intensive docking simulations, accelerating screening for large compound libraries. This flexibility, powered by generative AI, enables the framework to adapt to a wide range of diseases, including novel pathogens, ensuring responsiveness to diverse health scenarios.

## Method

**Figure 1.**
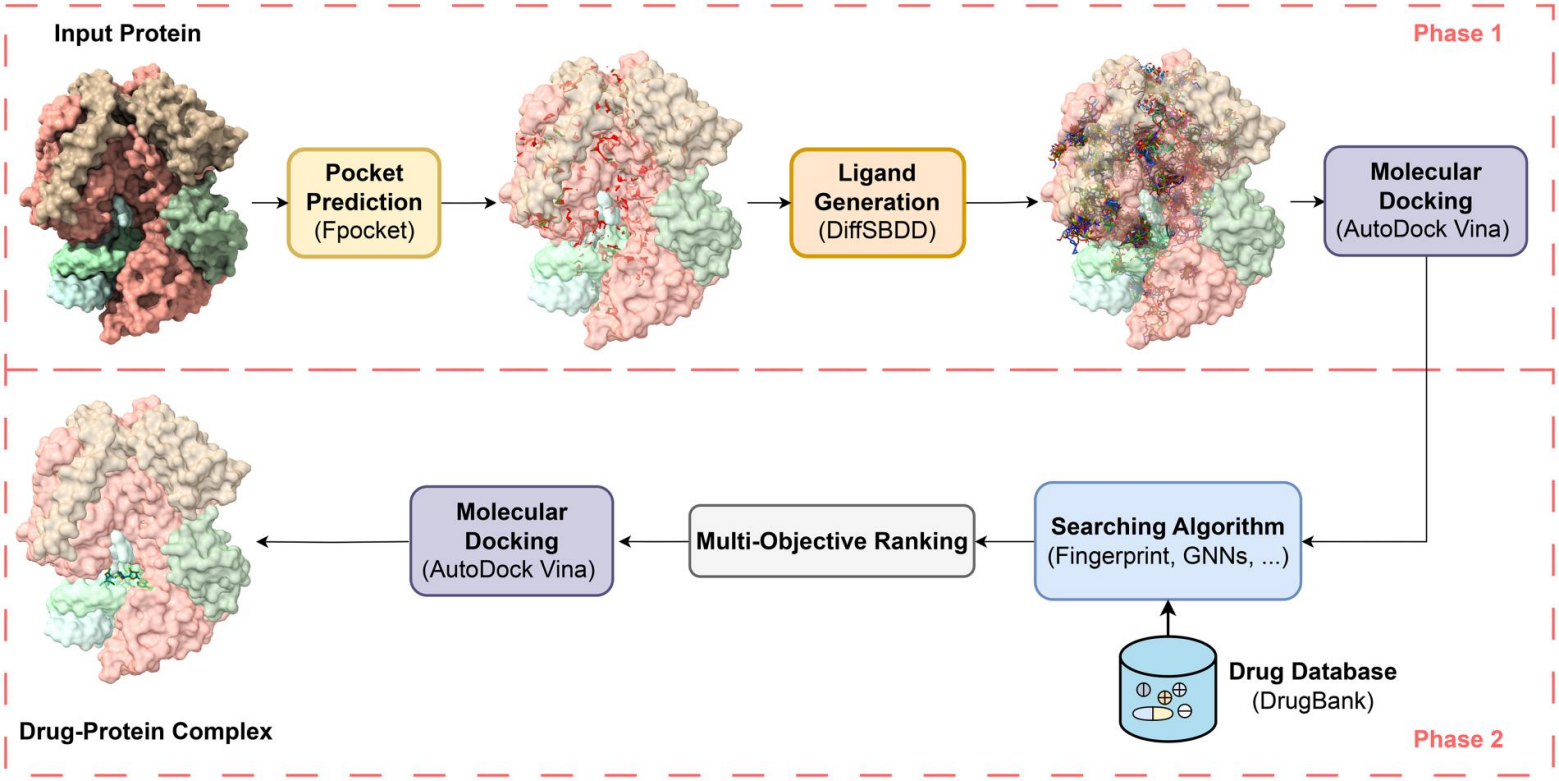
Overview of the generative AI-assisted drug repurposing pipeline. The pipeline consists of two phases: Phase 1 generates potential ligands using generative AI, and Phase 2 identifies promising drug candidates via similarity-based searches within drug databases.

The proposed pipeline (as illustrated in Fig. 1 and Algorithm 3) is structured into two distinct phases to enhance the efficiency and effectiveness of the drug repurposing process. In the first phase (as illustrated in Algorithm 1), potential binding pockets on the target protein are identified through advanced computational prediction methods. These predicted pockets are then used as specific input conditions for a generative AI model, which is employed to design novel ligand structures optimized for binding to these sites. The generated ligands are treated as initial drug candidates, thereby focusing subsequent screening efforts. In the second phase (as illustrated in Algorithm 2), the pipeline performs similarity searches within existing drug databases, comparing the geometric and chemical properties of the AI-generated ligands to those of approved drugs. This step operates on the foundational assumption that “chemical compounds with similar structures may have similar activities,” a principle that has been a cornerstone of lead identification in drug discovery for many years [11]. Compared to docking simulations, which can be computationally intensive and time-consuming, similarity search-based screening offers a significantly faster alternative, enabling the rapid identification of drug candidates while maintaining high relevance to the target protein.

### Phase 1—Potential ligand generation

Algorithm 1 Potential ligand generation

**Input:** protein

**Output:** ligand_generation

**Procedure**  phase1(protein)

 potential_pockets = ***PP***(protein)                                    ▹Pocket prediction

 **for each** *pocket* in potential_pockets **do**

  generative_ligands = ***LG***(*pocket*)                                   ▹Ligand generation

  **for each** *ligand* in generative_ligands **do**

   binding_energy = ***MD***(protein, *ligand*, *pocket*)                            ▹Molecular docking

   ligand_generation.add(*ligand*, *pocket*, binding_energy)

  **end for**

 **end for**

 **return** ligand_generation

**end procedure**

#### Pocket prediction

We applied Fpocket [24] as a tool for identifying and characterizing protein pockets, which are potential binding sites for small molecules. It works by detecting cavities on the protein’s surface and then analyzing these cavities to identify important features such as size, volume, and hydrophobicity. These pockets are often crucial for drug design, as they can serve as target sites for inhibitors or other therapeutic compounds.

#### Ligand generation

After identifying the potential binding pockets using Fpocket [24], we employed DiffSBDD (Structure-based Drug Design with Equivariant Diffusion Models) [16] to design candidate molecules that can effectively bind to these pockets. DiffSBDD leverages the 3D structure of the protein and its identified pockets to guide the design of ligands that fit optimally into the binding site, improving binding affinity and specificity. The equivariant diffusion models play a critical role by maintaining the geometric and symmetrical properties of molecular structures throughout the generative process, ensuring that the designed molecules conform to the physical constraints of the protein–ligand interactions. These models help generate diverse, high-quality candidate molecules by iteratively refining molecular structures to optimize their fit within the pocket. This approach integrates both the structural information of the protein and the physical properties of potential drug-like molecules, enhancing the efficiency and accuracy of drug discovery efforts.

#### Molecular docking of generative ligands

To calculate the BE of generative ligands, we use Vina Docking 3D [25, 26], a molecular docking tool, to simulate and predict how well these ligands interact with the identified binding pockets of the protein. Vina works by exploring possible orientations and conformations of the ligand within the binding site and calculating the binding affinity based on molecular forces such as van der Waals interactions, hydrogen bonding, and electrostatic interactions. By running multiple docking simulations, Vina predicts the most favorable binding pose of the ligand and provides a BE score, which reflects the stability and strength of the ligand–protein interaction. This score helps prioritize the best candidates for further optimization or experimental validation, streamlining the process of identifying high-affinity drug candidates in structure-based drug design.

### Phase 2—Potential drug searching

Algorithm 2 Potential drug searching

**Input:** protein, ligand_generation, drug_database

**Output:** potential_ligands

**Procedure**  phase2(protein, ligand_generation, drug_database)

 potential_ligands = ∅

 **for each** *drug* in drug_database **do**

  sligand, spocket, sbinding_energy = ***SA***(*drug*, ligand_generation)                    ▹ Search algorithm

  drug_database[drug].add(sligand, spocket, sbinding_energy)

 **end for**

 ranked_drug_database = ***MR***(drug_database)                           ▹ Multi-objective ranking

 **for topk** *drug*, *spocket* in ranked_drug_database **do**

  3d_poses, drug_binding_energies = ***MD***(protein, *drug*, *spocket*)                     ▹ Molecular docking

  potential_ligands.add(*drug*, 3d_poses, drug_binding_energies)

 **end for**

 **return** ligand_generation

 **end procedure**

#### Searching algorithm

To identify structurally or functionally similar compounds from drug databases, we explored multiple molecular encoding methods to map both generated ligands and existing compounds into a continuous embedding space. Specifically, we evaluated the effectiveness of Tanimoto similarity [27], Morgan fingerprint similarity [28], Graph Neural Networks (GNNs) [29], Graph Attention Networks (GATs) [30], Equiformer [31], and Equivariant Graph Neural Networks (EGNNs) [32]. Background information and technical details of these models are provided in Appendix A.1. For all methods except Tanimoto similarity, we compute cosine similarity between the embedding vectors of generated ligands and compounds in the drug database to assess molecular similarity. In the case of Tanimoto similarity, we use binary Morgan fingerprints and apply the standard Tanimoto coefficient (Jaccard index) as the similarity metric. This unified framework enables the identification of potential analogs or repurposable compounds based on their structural representations in the latent space. For each drug in the database, we assign the most similar generative ligand based on the chosen similarity score (SS) to estimate its likely BE and predicted pocket coordinates.

#### Multi-objective ranking

The rank-sum weight method [33, 34] is employed to rank drugs by considering both their structural similarity to generative ligands and their BE, ensuring a balanced evaluation of multiple criteria. The ranks assigned to each criterion are summed to produce a final score, with optional weighting to emphasize the relative importance of each factor. Drugs with the highest total rank-sum values are prioritized, as they exhibit strong performance in both BE and structural similarity.

The fitness function, formulated as:

(1) $$f(x)=\frac{w_{1}\times f_{1}(x)}{\sqrt{E(f_{1}{\left(x\right)}^{2})}}-\frac{w_{2}\times f_{2}(x)}{\sqrt{E(f_{2}{\left(x\right)}^{2})}},$$

 

(2) $$w_{i}=\frac{2(n+1-i)}{n(n+1)},$$

assigns a score to each drug *x* in the database based on the rank–sum criterion. Here, $f_{1}(x)$ represents the SS between *x* and the most similar ligand in the generative ligand database, while $f_{2}(x)$ corresponds to the BE of that ligand. The expectation operator *E* ensures normalization across the dataset, making the scores comparable. The weighting scheme $w_{i}$ prioritizes higher-ranked drugs by assigning greater influence to lower rank values.

The resulting score provides a quantitative measure for drug ranking, with higher values indicating better candidates. The best-performing drugs exhibit high SSs (strong structural resemblance to generative ligands) and low binding energies (strong interactions with the target protein), maximizing f(x). Conversely, drugs with weak similarity or poor binding energies receive lower scores, placing them lower in the ranking.

#### Molecular docking of top-ranked drugs

After generating a ranked list of candidate drugs based on their structural similarity to AI-generated ligands, we apply molecular docking to evaluate the binding potential of the top-ranked compounds. This step complements the similarity-based retrieval by providing 3D structural insight into how each candidate might interact with the target protein. In contrast to Phase 1, where docking is used to assess the generated ligands, this phase focuses on real drugs from the database to refine and interpret the most promising candidates.

Each top-ranked drug is assigned a predicted binding pocket based on its most similar generative ligand, and molecular docking is performed to generate candidate binding poses and energy scores. This allows DrugPipe to not only prioritize compounds based on their structural similarity but also to assess their physical compatibility with the protein target. The resulting drug–target complexes provide interpretable 3D models that may inform downstream validation and hypothesis generation. More broadly, this component of the pipeline serves two key purposes: (i) to simulate the real-world screening effort required to identify a viable binder and (ii) to enable structural comparison against known drug–target complexes when such references are available. In cases where experimental structures exist, docking predictions can be evaluated qualitatively; otherwise, they offer plausible hypotheses for future validation.

Algorithm 3 Generative AI-assisted drug repurposing pipeline

**Input:** protein, drug_database

**Output:** potential_ligands

**Procedure**  pipeline(protein, drug_database)

 ligand_generation = ***PHASE1***(protein)

 potential_ligands = ***PHASE2***(protein, ligand_generation, drug_database)

 **return** potential_ligands

**end procedure**

### Experiment

#### Evaluation on drug repurposing scenarios

To evaluate the generalizability and practical utility of ‘DrugPipe,’ we conducted a comprehensive assessment using the DrugBank drug–target interaction dataset [35], which contains 846 protein targets and 9716 approved drugs. This evaluation simulates realistic drug repurposing scenarios, where the objective is to identify existing drugs that could potentially bind to novel or poorly characterized protein targets, such as those associated with emerging diseases like COVID-19 and HIV.

The primary goal is to determine how effectively DrugPipe can prioritize clinically approved drugs using only structural information, without relying on prior knowledge of known binders. Since DrugPipe ranks compounds based on similarity to generative ligands, we hypothesize that approved drugs with true binding potential will be ranked near the top, despite the absence of supervised training data. We acknowledge, however, that not all approved drugs are optimized for binding affinity alone because factors such as pharmacokinetics, toxicity, and off-target effects also influence their clinical viability. To account for this, we evaluate performance by reporting the distribution of the rank at which the first approved drug appears for each target. This metric reflects how well the pipeline enriches clinically relevant compounds and narrows the experimental search space.

To contextualize these results, we include a benchmark comparison against traditional blind docking. This experiment is designed to assess how DrugPipe compares to conventional virtual screening approaches that do not incorporate enrichment. Specifically, we use QVina-W [36] to perform exhaustive docking of all approved DrugBank compounds against four selected protein targets. These include two widely studied targets from major disease areas, such as HIV reverse transcriptase and SARS-CoV-2 RNA-dependent RNA polymerase; as well as two representative targets from DrugBank: penicillin-binding protein 1a (PBP1a) and human pancreatic alpha-amylase (HPA). Due to the significant computational cost of docking across the full DrugBank and target space, we restrict this benchmark to four case studies selected for their biomedical relevance and structural diversity. Unlike DrugPipe, which filters and ranks candidates prior to docking, blind docking performs no prior enrichment or pocket conditioning. Compounds are ranked solely by their docking scores, and we evaluate how early approved drugs appear in the output. This setup enables a direct comparison of hit rate and run-time between DrugPipe and traditional docking-based pipelines.

In addition to ranking performance, we also compare the predicted 3D drug–target complex structures generated by DrugPipe and blind docking with experimentally resolved complexes for these case studies. This qualitative analysis helps assess not only whether approved drugs are retrieved, but also whether their predicted binding modes align with known structural data. Finally, it is worth noting that compounds ranked above approved drugs by either method may represent novel but previously untested candidates. As such, this evaluation not only highlights DrugPipe’s ability to recover validated drugs efficiently, but also underscores its potential to suggest new leads for downstream investigation, contributing to a more scalable and practical approach to structure-based drug repurposing.

#### Ablation study

To assess the robustness and generalizability of our generative AI-assisted drug repurposing pipeline, we conducted an ablation study focused on three key objectives: (i) evaluating the effectiveness of AI-generated ligands as surrogates for known drugs in similarity-based searches, (ii) comparing the performance of various molecular encoding methods for similarity-based retrieval, and (iii) assessing the impact of multi-objective ranking on candidate selection. These experiments were designed to test the pipeline under different configurations and assumptions. The first study addresses a critical scenario, that is, novel targets with no known ligands, by examining whether generated compounds can guide similarity search as effectively as real drugs. The second benchmark tests several encoding techniques to identify the most effective representation for compound similarity. The third explores whether integrating both similarity and binding affinity scores into a multi-objective ranking improves screening efficiency compared to single-objective approaches.

#### AI-generated ligands as surrogates for known drugs

In the first part of the study, we assess whether AI-generated ligands can effectively serve as surrogates for known drugs in similarity-based search. To establish a baseline, we randomly selected one approved drug per target from the DrugBank dataset and used it as the query molecule to retrieve similar compounds from the drug database. We then compared the baseline results with the outcomes obtained when using AI-generated ligands as query compounds in the similarity-based search. This comparison allows us to evaluate how well generative models can emulate real drugs in guiding the search for potential repurposing candidates, particularly in data-scarce settings where no known ligands exist for a novel target.

#### Comparison of molecular encoding methods

We evaluated six molecular encoding techniques, such as Tanimoto similarity, Morgan fingerprint similarity, GNNs, GATs, Equiformer, and EGNNs to determine how effectively they support similarity search in the context of drug repurposing. All encoding methods were tested using a unified multi-objective ranking framework to ensure consistency. The comparison focused on two metrics: hit rate and computational cost. Hit rate quantifies how well each method ranks approved drugs near the top of the similarity list, reducing the number of compounds requiring further evaluation. A higher hit rate indicates better prioritization and a more efficient narrowing of the search space. Computational cost was measured as the average time required to compute similarity rankings across the entire drug database for all protein targets. This reflects the practical run-time efficiency of each method. Since the most accurate approach may not always be the most scalable, the goal was to identify a method that provides the best trade-off between accuracy (hit rate) and efficiency (run-time) for large-scale drug repurposing scenarios.

#### Multi-objective ranking

In the second part of our ablation study, we evaluated the impact of different ranking strategies on the efficiency of downstream docking and the recovery of approved drugs. We compared single-objective rankings based solely on either structural similarity or predicted BE against a multi-objective ranking strategy that combines both. All strategies were applied using the GNN-based molecular encoder, which offered the best trade-off between accuracy and run-time in our similarity search evaluation (see “Results, Ablation study results”). To assess the practical utility of each ranking method, we simulate a real-world screening process where compounds are docked sequentially until a validated hit is recovered. In practice, the number of top-*k* compounds tested is not fixed in advance; researchers typically proceed iteratively until a binder is identified. In our controlled setting, however, we leverage ground-truth labels and define *k* as the rank position of the first known approved drug for each target. We then perform molecular docking on all top-*k* compounds and record the total run-time required to reach the approved drug. This provides a standardized measure of efficiency and allows us to quantify the resource cost associated with each ranking strategy. Additionally, when experimentally determined drug–target complex structures are available, we compare the predicted docking pose of the approved drug to its known binding conformation. This qualitative analysis helps assess the structural plausibility of the predictions. For top-ranked compounds without reference complexes, we do not perform further evaluation, though these remain viable candidates for future experimental validation. To reduce computational burden while preserving representativeness, this experiment was conducted on a 10% subset of the DrugBank dataset, stratified to maintain the original distribution of compound–target pairs. This analysis ultimately highlights whether combining multiple objectives in the ranking process can improve both hit retrieval and docking efficiency, which are key factors for real-world applicability in large-scale drug repurposing workflows.

## Results

This section presents the performance evaluation of DrugPipe. We first demonstrate the pipeline’s effectiveness in drug repurposing scenarios using the DrugBank dataset, followed by an ablation study to assess the contribution of individual components. Given that DrugPipe is modular and configurable, the ablation study serves to validate the design decisions and identify the most efficient configuration for broader application. For reference, the evaluation metrics are described in detail in “Metrics and description” section, while the experimental setup, including computational resources, software versions, and dataset information, is provided in “Experimental details” section Additional results that further support our findings can be found in “Additional results” section.

### Drug repurposing evaluation results

**Figure 2.**
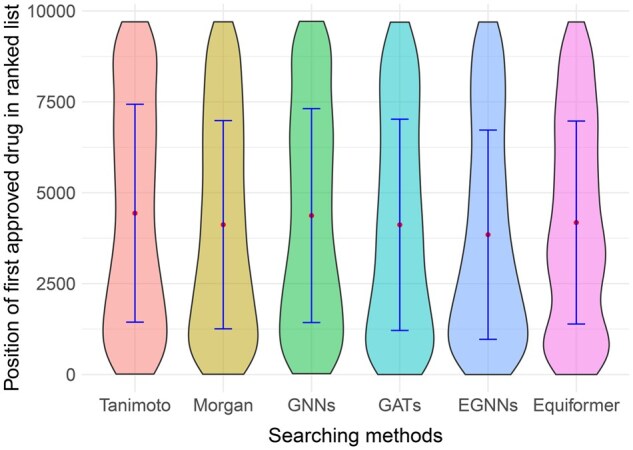
Distribution of the rank positions of the first approved drug for each protein target in the DrugBank DTI dataset across different molecular encoding methods (as mentioned in “Phase 2—Potential drug searching”).

In this section, we assess the performance of DrugPipe on the DrugBank drug–target interaction dataset, which includes 846 protein targets and 9716 approved drugs. This evaluation simulates a challenging repurposing scenario in which no prior ligand information is available for the targets. As shown in Fig. 2, DrugPipe consistently ranks clinically approved drugs toward the top of the candidate list across various molecular encoding strategies. Focusing on the GNN-based model, which serves as the optimal molecular representation method (see “Results, Ablation study results” section for details), we observe a median rank of approximately 2100, with the first quartile (Q1) around 900 and the third quartile (Q3) near 4500. These results indicate that DrugPipe substantially narrows the candidate space, frequently surfacing approved drugs within the top 20%–30% of the ranked list. This level of prioritization is particularly encouraging given the zero-shot setting, where no known ligands or pocket annotations are used.

To further illustrate its practical effectiveness, we highlight several representative case studies. For the HPA target, DrugPipe ranked Acarbose, a known inhibitor, at position 253. For PBP1a; Cefoperazone, a beta-lactam antibiotic, was ranked at position 52. In viral applications, Abacavir, an antiretroviral targeting HIV reverse transcriptase [37], was retrieved at position 1055, while Remdesivir, which targets severe acute respiratory syndrome coronavirus 2 (SARS-CoV-2) RNA-dependent RNA polymerase [38], appeared at position 637. These examples demonstrate that DrugPipe can effectively recover clinically validated drugs across a range of therapeutic targets.

To provide a more rigorous benchmark, we compared DrugPipe with QVina-W, a blind docking approach applied to the same set of DrugBank compounds for each of the four selected targets. As shown in Table 2, DrugPipe achieved comparable or superior hit rate reduction in three out of four cases while consistently demonstrating substantial improvements in computational efficiency. For example, on the HPA and PBP1a targets, DrugPipe not only retrieved the approved drugs more effectively than QVina-W but also completed the docking in significantly less time. While QVina-W slightly outperformed DrugPipe in the HIV-1 case in terms of hit rate, DrugPipe still offered a clear run-time advantage. Overall, the speedup of DrugPipe over QVina-W ranged from 1.5× to nearly 7× across all tested scenarios, underscoring its efficiency in large-scale virtual screening.

**Table 2.** Comparison of QVina-W blind docking and DrugPipe on representative drug repurposing targets, evaluated in terms of hit rate reduction and total run-time.

Protein	Hit rate reduction (%)		Total run-time (hours)	
–	QVina-W	DrugPipe	QVina-W	DrugPipe
SARS-CoV-2	60.41	**93.44**	48.59	**19.29** (2.5×)
HIV-1	**98.90**	89.14	73.05	**49.60** (1.5×)
PBP1a	95.98	**99.46**	38.63	**20.56** (1.9×)
HPA	83.84	**97.40**	52.32	**7.71** (6.8×)

Speedup rates for DrugPipe, relative to QVina-W, are shown in parentheses alongside the run-time values.

*Note:* Bold values indicate better performance for the corresponding metric.

**Figure 3.**
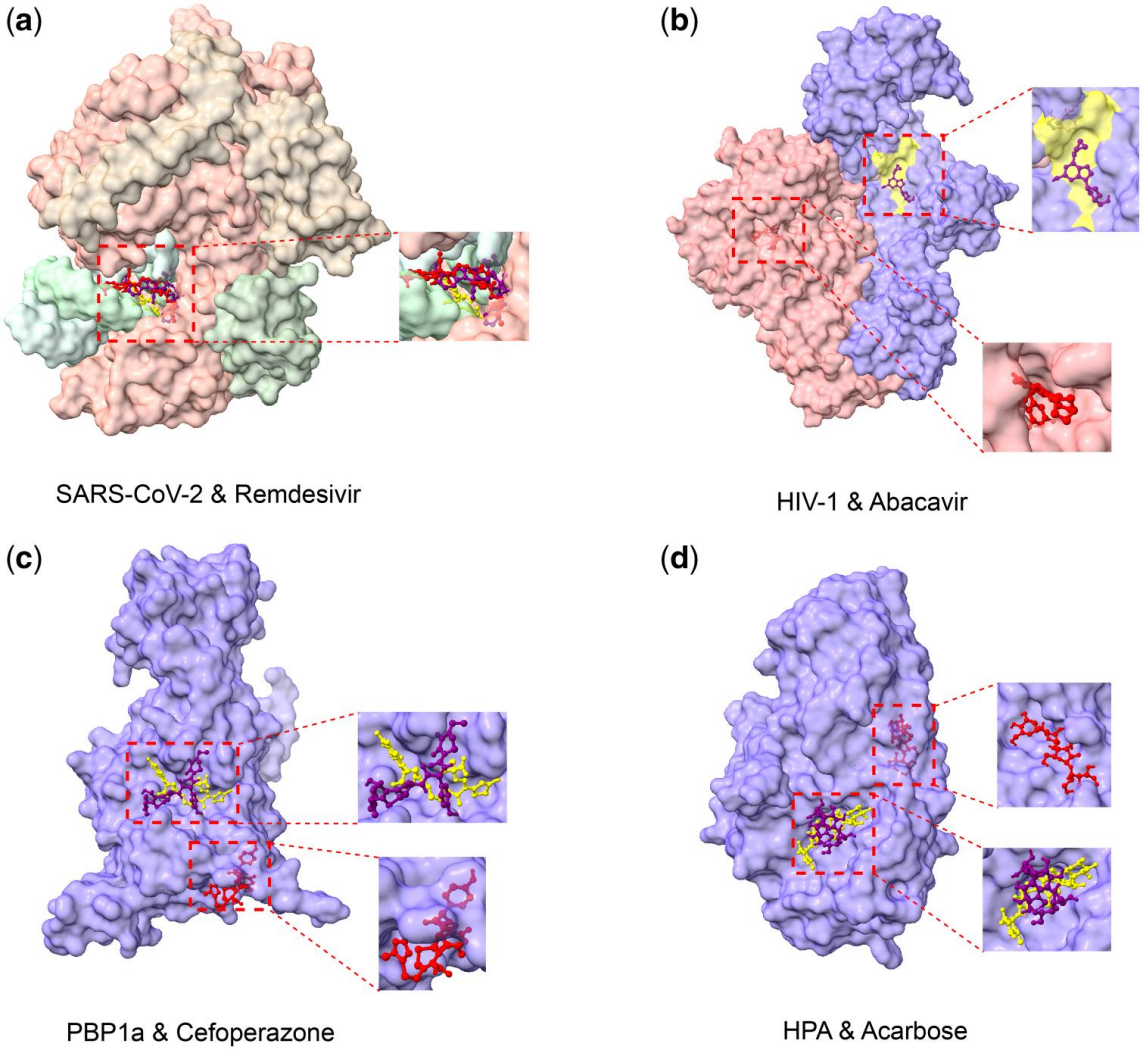
Case studies comparing drug–target complex conformations. For each pair, we overlay the DrugPipe-predicted pose (purple), the blind docking result from QVina-W (red), and the experimentally determined pose (yellow). The examples shown are: (a) SARS-CoV-2 RNA-dependent RNA polymerase with Remdesivir, (b) HIV-1 reverse transcriptase with Abacavir, (c) PBP1a with Cefoperazone, and (d) HPA with Acarbose. The figure illustrates how DrugPipe’s binding predictions align with known structures and differ from traditional blind docking approaches.

Beyond ranking performance, DrugPipe also generates predicted 3D drug–target complex conformations, offering structural insight into potential binding interactions. As shown in Fig. 3, we visualize and compare the predicted binding poses from DrugPipe, the corresponding poses generated by QVina-W blind docking, and the experimentally determined structures for selected case studies. Despite operating without prior knowledge of known binding pockets, DrugPipe often produces conformations that are more localized to plausible binding regions and better aligned with experimentally validated complexes. These observations underscore a key challenge in blind virtual screening: even standard tools like QVina-W, when applied without prior pocket information, can sometimes yield suboptimal or misplaced binding poses. In contrast, DrugPipe leverages predicted pockets, conditioned on generative ligands, to guide docking toward more realistic binding sites. While structural accuracy varies across targets, the ability to produce interpretable and contextually consistent 3D poses reinforces DrugPipe’s utility as a practical and effective framework for structure-based drug repurposing.

### Ablation study results

**Figure 4.**
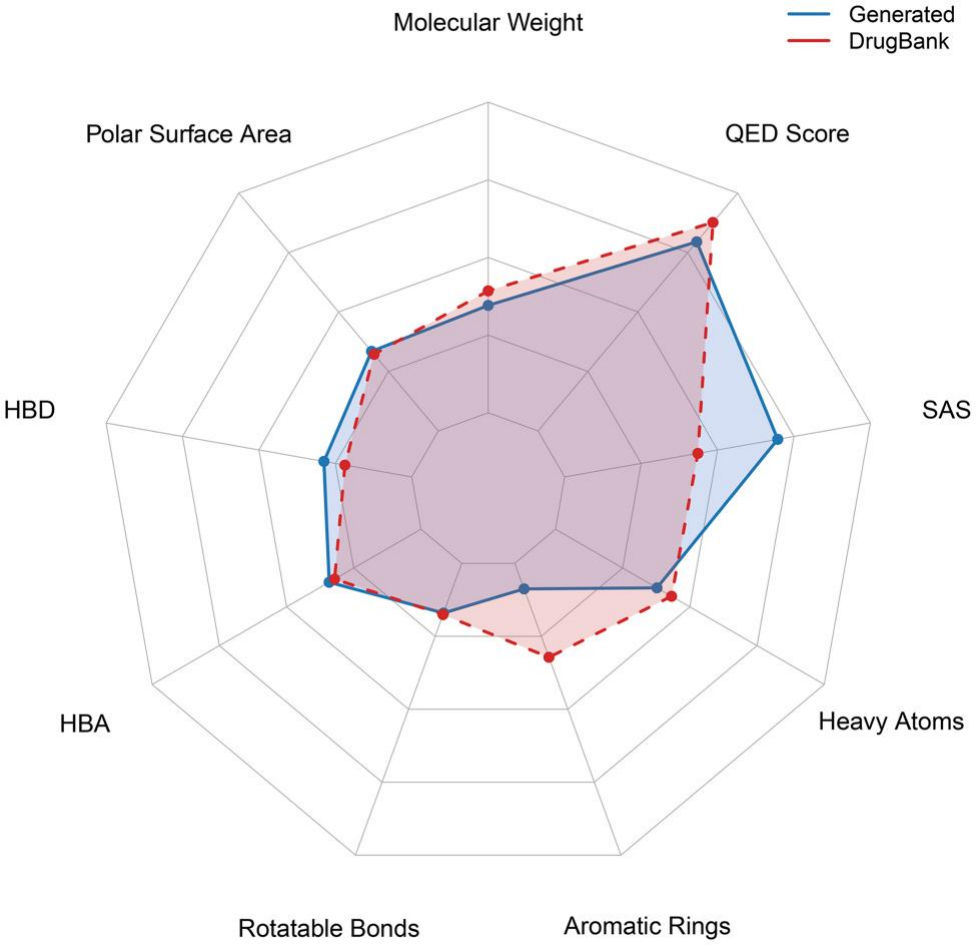
Comparison of physicochemical and drug-likeness properties between AI-generated ligands and DrugBank compounds. The chart includes the following properties: molecular weight (overall molecular size), QED Score (quantitative estimate of drug-likeness), SAS (synthetic accessibility score), heavy atoms (count of non-hydrogen atoms), aromatic rings (number of aromatic ring systems), rotatable bonds (measure of molecular flexibility), HBA/HBD (hydrogen bond acceptors/donors), and polar surface area (related to solubility and permeability). This comparison highlights structural and chemical differences that may impact drug-likeness and downstream screening performance.

We evaluated the performance of various molecular encoding methods using two types of query compounds: AI-generated ligands and real approved drugs. The results, summarized in Table 3, reveal a consistent trend: real drugs outperform AI-generated ligands across all encoding methods in terms of hit rate reduction. For instance, GNNs achieved a hit rate reduction of 92.88% when using real drugs, which dropped significantly to 55.76% when using AI-generated ligands. These findings highlight a key limitation of current generative models: although they can produce molecules with high predicted binding affinities, the generated structures often diverge from real, synthesizable drugs in terms of chemical properties and drug-likeness. As illustrated in Fig. 4, AI-generated ligands tend to deviate from DrugBank compounds across several drug-likeness criteria, including Quantitative Estimate of Drug-likeness (QED) score, synthetic accessibility, and key molecular properties such as molecular weight and polar surface area. These discrepancies contribute to their reduced effectiveness in downstream similarity-based retrieval, as the generated compounds may not adequately reflect the structural and pharmacokinetic features of approved drugs. Nonetheless, generative ligands may still offer value in broadening chemical search space, especially for novel targets with no known ligands. While additional filtering, optimization, or refinement strategies could potentially improve their downstream utility, systematically designing and evaluating such post-processing mechanisms remains a nontrivial task and is left as an important direction for future work.

**Table 3.** Comparison of molecular encoding methods for hit rate reduction using AI-generated ligands and real drugs.

Method	Hit rate reduction (%)		Run-time (S)
–	AI-generated ligands	Real drugs	
Tanimoto	55.28	**95.62**	714.40
Morgan FP	58.12	95.47	786.98
GNN	55.76	92.88	**12.95**
GAT	58.36	92.57	13.86
EGNN	**60.67**	90.69	50.77
Equiformer	57.31	90.73	83.24

Run-time refers to the average search time per target.

*Note:* Bold values indicate better performance for the corresponding metric.

In comparing the molecular encoding methods under the AI-generated ligand setting, we observe that EGNNs achieved the highest hit rate reduction (60.67%), indicating strong potential for prioritizing relevant drug–target interactions even when starting from synthetic queries. Among the graph-based models, GATs also performed competitively, achieving a hit rate reduction of 58.36% with a relatively low run-time of 13.86 s. While GNNs had a slightly lower hit rate reduction (55.76%), they offered the best computational efficiency, with the shortest run-time at just 12.95 s. In contrast, traditional methods such as Tanimoto and Morgan Fingerprint similarity showed comparable or lower performance (55.28% and 58.12%, respectively) but required significantly longer run-times (714.40 and 786.98 s). These results suggest that modern graph-based encoders, particularly EGNNs, are more robust when handling the noisier and less drug-like structures produced by generative models. However, when using real drugs as query compounds, the trend shifts. Traditional fingerprint-based methods, such as Tanimoto and Morgan fingerprints, outperform graph-based methods, achieving hit rate reductions of 95.62% and 95.47%, respectively. This likely reflects the fact that real drugs, being well-structured and drug-like, align better with the assumptions of fingerprint-based similarity metrics. In summary, while EGNNs offer the best hit rate performance, their significantly higher run-time limits their practicality for large-scale screening. GNNs, on the other hand, strike a more favorable balance between accuracy and efficiency, making them the most suitable choice for use in the main pipeline configuration. This decision reflects a trade-off between optimal performance and computational scalability, which we consider critical for real-world applications of DrugPipe.

In the multi-objective ranking study, we assessed the impact of combining SSs and BE as compared to using either criterion alone. As detailed in Table 4, the multi-objective approach yielded the best performance, with a hit rate reduction of 61.24%, outperforming both single-objective methods. While the SS + BE approach increased the docking time to 49.97 s, this is a reasonable trade-off given the significant improvement in ranking accuracy. The single-objective ranking based on BE alone was the fastest method, with an average docking time of 48.65 s, but it came at the cost of reduced hit rate performance (55.74%). This demonstrates that the multi-objective ranking strategy provides a balanced approach, optimizing both hit rate and computational efficiency in the drug repurposing pipeline.

**Table 4.** Comparison of single-objective and multi-objective ranking methods on the DrugBank DTI dataset.

Ranking method	Hit rate reduction (%)	Docking time (s)
SS	59.33	52.61
BE	55.74	**48.65**
SS + BE	**61.24**	49.97

*Note:* Bold values indicate better performance for the corresponding metric.

## Discussion

Blind virtual screening, in which no prior information about known ligands or binding pockets is available, presents a particularly challenging setting for drug repurposing. In this context, DrugPipe operates without access to experimentally validated reference compounds, relying instead on predicted pockets and generative ligands to guide candidate retrieval. While this enables a fully structure-based and target-agnostic workflow, it also introduces greater uncertainty into the screening process. Our evaluation confirms that performance under this setting is less robust than traditional workflows that leverage known actives. In particular, substituting AI-generated ligands with real approved drugs in the similarity search stage yields significantly better hit rate reduction, illustrating the current limitations of generative models in producing chemically and structurally realistic surrogates for known drugs.

Despite this inherent difficulty, DrugPipe demonstrates strong performance when compared to a conventional blind docking baseline. Across four diverse targets, including those associated with COVID-19 and HIV, DrugPipe consistently achieves comparable or superior hit rate reduction, while requiring substantially less computational time. Although the blind docking experiments were limited to a small number of targets due to their high resource demands, the results are representative and support the pipeline’s efficiency and effectiveness in practical repurposing scenarios. Beyond ranking, DrugPipe also generates interpretable 3D drug–target complex conformations. In several cases, these predicted poses more closely aligned with experimentally determined binding modes than those generated by blind docking. This suggests that the use of predicted pockets, even when inferred from generative ligands, can guide docking toward more realistic and biologically relevant interactions.

Importantly, DrugPipe is proposed as a modular and extensible pipeline rather than a fixed model. Each component from ligand generation and pocket prediction to similarity search and docking is designed to be replaceable and configurable. The current implementation reflects one instantiation of this framework, using specific models and tools selected to demonstrate the pipeline’s feasibility. However, future studies can readily substitute these components with more advanced or task-specific alternatives, enabling users to tailor the pipeline to different targets, data availability, or computational constraints. This flexibility ensures that DrugPipe is not only scalable and efficient for current blind virtual screening tasks but also adaptable to future advances in molecular generation, representation learning, and structure-based prediction. As such, it provides a forward-compatible foundation for continued innovation in in silico drug repurposing.

## Conclusion

We presented ‘DrugPipe,’ a Generative AI-assisted Virtual Screening Pipeline that enables efficient, scalable, and generalizable *in silico* drug repurposing. By integrating binding pocket prediction, generative ligand modeling, and similarity-based retrieval from drug databases, ‘DrugPipe’ supports blind virtual screening for any protein target without requiring prior structural or functional knowledge, making it particularly valuable for novel, understudied, or emerging targets. Our evaluation using the DrugBank dataset, including case studies on HIV and COVID-19, demonstrated that ‘DrugPipe’ can effectively prioritize approved drugs while substantially reducing computational cost compared to standard docking methods such as QVina-W. We further explored the use of AI-generated ligands as query surrogates in similarity-based searches and found that, while they do not yet outperform real drugs, they offer a promising avenue for initiating repurposing in data-scarce scenarios. Although a gap remains between theoretical binding predictions and real-world drug-likeness, ‘DrugPipe’s’ modular architecture ensures adaptability to future advances in generative modeling and virtual screening. These qualities position it as a practical and extensible tool for accelerating drug repurposing efforts across a wide range of therapeutic areas.

## Authors’ contributions

Phuc Pham [Data curation (equal), Formal analysis (equal), Software (equal), Validation (equal), Visualization (equal), Writing—original draft (equal), Writing—review & editing (equal)], Viet Thanh Duy Nguyen [Data curation (equal), Formal analysis (equal), Software (equal), Validation (equal), Visualization (equal), Writing—original draft (equal), Writing—review & editing (equal)], and Kyu Hong Cho [Conceptualization (equal), Investigation (equal), Methodology (equal), Project administration (equal), Supervision (equal), Validation (equal), Writing—original draft (equal), Writing—review & editing (equal)], Truong Son Hy [Conceptualization (lead), Funding acquisition (lead), Investigation (lead), Methodology (lead), Project administration (lead), Resources (lead), Software (equal), Supervision (equal), Writing—original draft (equal), Writing—review & editing (equal)].

*Conflict of interest statement*. The authors declare that they have no conflict of interest.

## Funding

Not applicable.

## Figures and Tables

**Figure A1.**
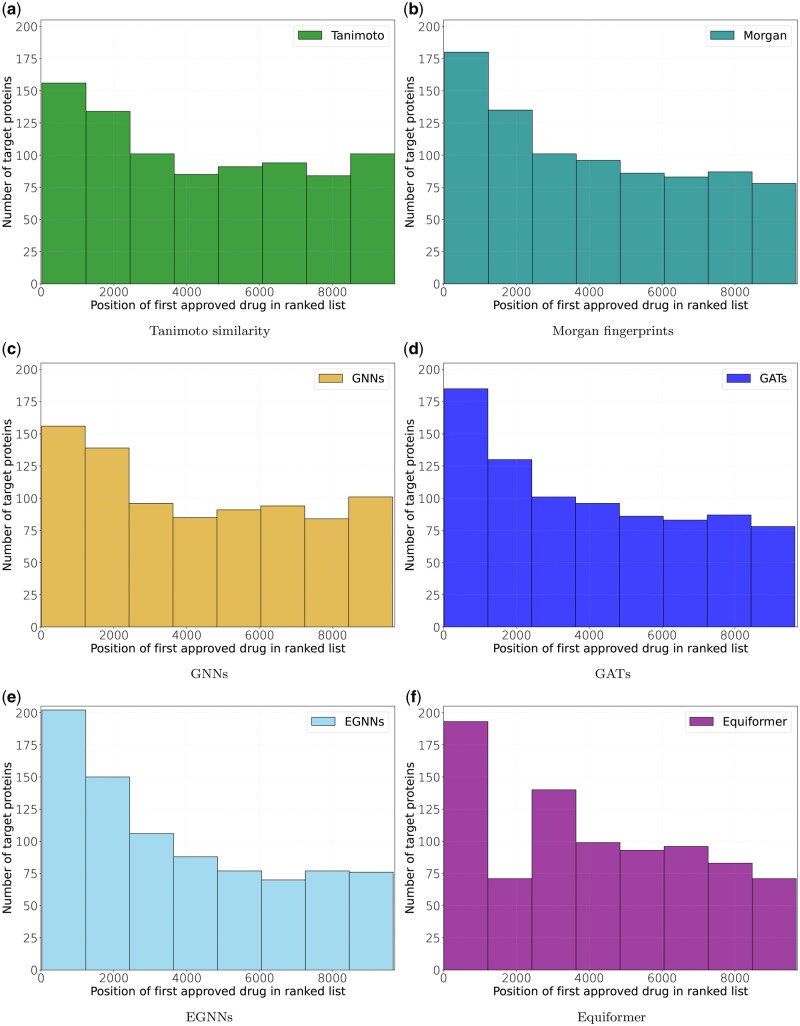
Distribution of protein targets based on the ranking position of their first approved drug in the DrugBank DTI dataset. The x-axis represents the rank position of the first approved drug (ranging from 0 to 9717), while the y-axis shows the number of protein targets associated with each rank. This visualization highlights the effectiveness of various searching methods in prioritizing approved drugs across the dataset.

## Data Availability

The datasets analyzed during the current study are available in the DrugPipe repository: https://github.com/HySonLab/DrugPipe.

## Appendix A

### A.1 Background

#### A.1.1. Tanimoto similarity

The Tanimoto similarity [27], also known as the Tanimoto coefficient, is a widely used measure in cheminformatics and molecular informatics to compare the similarity between chemical compounds [39, 40]. It is particularly useful for comparing molecular fingerprints, which are binary vectors representing the presence or absence of certain molecular features.

In the context of Tanimoto, the attributes of set F will be the chemical elements. Define the n×m matrix $A=\{a_{ij}\}$ so that:

(A.1) $$\{\begin{array}{c} a_{ij}=1,\;\text{if}\;d_{i}\;\text{possesses}\;\text{the}\;\text{chemical}\;\text{element}\;f_{j}\\ a_{ij}=0,\;\text{otherwise} \end{array}$$

And g×m matrix $B=\{b_{ij}\}$ so that $b_{ij}$ is 1, if $l_{i}$ possesses the chemical element $f_{j}$, $b_{ij}$ is 0 if $l_{i}$ does not possess the chemical element $f_{j}$. The Tanimoto coefficient formula can be represented as:

(A.2) $$\sigma (d_{i},l_{j})=\frac{\left|f_{da}(d_{i})\cap f_{lb}(l_{j})\right|}{\left|f_{da}(d_{i})\cup f_{lb}(l_{j})\right|},$$

where $f_{da}:M\to {\left\{0,1\right\}}^{m}$ is a mapping that maps a compound $d_{i}$ in D to a row $a_{i}$ of matrix *A*, $f_{lb}:M\to {\left\{0,1\right\}}^{m}$ is a mapping that maps a compound $l_{i}$ in L to a row $b_{i}$ of matrix *B*, with M is the space containing all compounds in the form of Simplified Molecular-Input Line-Entry System [41].

Let us denote $f_{da}(d_{i})\cap f_{lb}(l_{i})$, $f_{da}(d_{i})\cup f_{lb}(l_{i})$ are m-dimensional row vectors in such a way that if $\alpha_{i}^{u}$ and $\alpha_{j}^{u}$ are the $u^{th}$ component of $f_{da}(d_{i})$ and $f_{lb}(l_{i})$ respectively, the $u^{th}$ component of $f_{da}(d_{i})\cap f_{lb}(l_{i})=\alpha_{i}^{u}\cdot \alpha_{j}^{u}$ and $f_{da}(d_{i})\cup f_{lb}(l_{i})=\alpha_{i}^{u}+\alpha_{j}^{u}+\alpha_{i}^{u}\cdot \alpha_{j}^{u}$ (mod 2). The numerator and the denominator of $\sigma (d_{i},l_{j})$ are the number of ones in the vector $f_{da}(d_{i})\cap f_{lb}(l_{i})$ and $f_{da}(d_{i})\cup f_{lb}(l_{i})$, respectively.

#### A.1.2. Morgan fingerprint

Morgan fingerprints [28], also known as circular fingerprints, are a type of molecular fingerprint used in cheminformatics to represent molecular structures. Morgan fingerprints are particularly popular in applications such as similarity searching, virtual screening, and machine learning tasks related to chemical informatics.

With Morgan fingerprint, the set F includes the hash values of the substructures with features defined using SMILES (Simplified Molecular-Input Line Entry System) arbitrary target specification (SMARTS) language [42] and is generated through the iterative updating stage [43] from the compound within a fixed radius. After creating the set of substructures, we calculate in the same way as the formula in “Background, Tanimoto similarity” to obtain the Morgan fingerprint similarity matrix.

#### A.1.3. GNN

Graphs are versatile data structures capable of representing intricate relational data, consisting of nodes and edges. They are prevalent across various fields, including social networks [44], computational chemistry [45–47], biology [48], recommendation systems [49], chip design [50], code understanding [51], semi-supervised learning [52], and more.

*Graph representation*. A graph G=(V,E) consists of a set of nodes *V* and edges *E*, where each node v∈V is associated with a feature vector $x_{v}$, and each edge (u,v)∈E represents a relationship between two nodes *u* and *v*. The graph structure is represented by an adjacency matrix *A*, where $A_{uv}=1$ if there is an edge between nodes *u* and *v*, and $A_{uv}=0$ otherwise.

*Message passing GNNs*. The initial GNN model introduced by [53, 54] was the pioneering deep learning approach for graph-structured data. It conceptualizes the task of graph embedding as a process of information diffusion, where nodes transmit information to their neighboring nodes, continuing until a stable equilibrium state is achieved. In each layer of a GNN, every node updates its feature vector by aggregating the information from its neighboring nodes. This is done through a message-passing mechanism, where messages are passed along the edges of the graph. The message-passing process typically involves two steps:

- **Aggregation**: For each node *v*, the feature vectors of its neighboring nodes N(v) are aggregated using a permutation-invariant function such as sum, mean, or max.
- **Update**: The aggregated message is then combined with the node’s current feature vector $h_{v}$ to produce an updated representation. This can be done through a learnable transformation, such as a neural network, often in the form of a linear transformation followed by a nonlinear activation function:
  (A.3) $$h_{v}^{\left(k+1\right)}=\sigma \left(W^{\left(k\right)}\cdot \text{Aggregate}(h_{u}^{\left(k\right)},\forall u\in N(v))\right),$$

where $h_{v}^{\left(k\right)}$ is the feature vector of node *v* at layer *k*, $W^{\left(k\right)}$ is a learnable weight matrix, σ is a nonlinear activation function (e.g., ReLU [55]), and the aggregation function computes a summary of the neighboring nodes’ features.

#### A.1.4 ENN

ENN are models designed to respect the symmetries present in the data, ensuring that certain transformations of the input result in predictable transformations of the output. This is particularly important in domains such as physics, chemistry, and computer vision, where data often exhibit intrinsic symmetries. In ENNs, these symmetries are encoded into the architecture, leading to models that generalize better, are more data-efficient, and respect physical laws. The two key types of equivariance discussed here are ‘permutation equivariance’ [56] and ‘rotation equivariance’ [57]. These correspond to different symmetries depending on the structure of the data.

*Permutation equivariance*. Permutation equivariance arises when the data is unordered, such as in graphs or sets. For example, molecular structures can be viewed as graphs, where nodes represent atoms and edges represent bonds, and permuting the atoms should not change the physical properties of the molecule. Formally, a function f:X→Y is permutation-equivariant with respect to the symmetric group $S_{n}$, which permutes the nodes of a graph. This means that for any permutation $\Pi \in S_{n}$, the function satisfies the property:

(A.4) f(Πx)=Πf(x),

where Π is an n×n permutation matrix, and $x\in R^{n\times d}$ is the input node feature matrix with *n* nodes and *d* features per node. This property ensures that if the input nodes are permuted, the output is permuted in the same way, making models like GNNs suitable for problems with unordered data. In a GNN, node features $h_{i}$ are updated by aggregating information from neighboring nodes:

(A.5) $$h_{i}^{\left(l+1\right)}=\sigma \left(W^{\left(l\right)}h_{i}^{\left(l\right)}+\sum_{j\in N(i)}A_{ij}\cdot W^{\left(l\right)}h_{j}^{\left(l\right)}\right),$$

where $h_{i}^{\left(l\right)}$ is the feature vector of node *i* at layer *l*, $A_{ij}$ is the adjacency matrix of the graph, $W^{\left(l\right)}$ are the learnable weights, and σ is a nonlinear activation function. This architecture respects permutation equivariance because permuting the nodes permutes both the features and the adjacency matrix, leaving the update rule unchanged.

*Rotation equivariance*. Rotation equivariance is relevant when the data reside in physical space and is subject to transformations such as rotations and reflections. For instance, in molecular modeling, atomic coordinates can be rotated or reflected, but the physical properties of the molecule should remain consistent under these transformations. A function *f* is said to be rotation-equivariant if applying a rotation or reflection to the input *x* results in an equivalent transformation of the output:

(A.6) f(Rx)=Rf(x),

where R∈O(3) is an orthogonal matrix representing a rotation or reflection in three-dimensional space. Here, $x\in R^{n\times 3}$ represents the 3D coordinates of *n* points (e.g., atoms), and f(x) might represent quantities like forces or velocities that should transform in the same way under rotations.

A common example of a rotation-equivariant network is an E(3)-equivariant network, where the goal is to respect transformations from the Euclidean group E(3), which includes rotations, translations, and reflections. In these networks, both node features (such as atomic types) and geometric coordinates (3D positions) are updated while preserving equivariance.

For instance, consider a feature update rule in an E(3)-equivariant network:

(A.7) $$h_{i}^{\left(l+1\right)}=\phi \left(h_{i}^{\left(l\right)},\sum_{j\in N(i)}\psi (h_{i}^{\left(l\right)},h_{j}^{\left(l\right)},d_{ij})\right),$$

where $d_{ij}=||x_{i}-x_{j}|{|}^{2}$ is the squared Euclidean distance between nodes *i* and *j*, and ϕ, ψ are learnable functions. For the coordinate updates, the equivariant transformation rule is applied:

(A.8) $$x_{i}^{\left(l+1\right)}=x_{i}^{\left(l\right)}+\sum_{j\in N(i)}\left(x_{i}^{\left(l\right)}-x_{j}^{\left(l\right)}\right)\cdot \phi (h_{i}^{\left(l\right)},h_{j}^{\left(l\right)},d_{ij}),$$

where the term $\left(x_{i}^{\left(l\right)}-x_{j}^{\left(l\right)}\right)$ ensures that the update respects the relative distances between the points, making it equivariant under rotations and translations.

In diffusion models, equivariance can also be applied to the distribution of data over time. A conditional distribution p(y|x) is said to be equivariant to a group *G* (e.g. the Euclidean group E(3)) if it satisfies:

(A.9) p(Ry|Rx)=p(y|x) for all R∈G,

where *x* and *y* could represent the input and output distributions over time, and *R* is a transformation such as rotation. This ensures that the diffusion process respects the symmetries of the data at every time step. In practice, this is useful in denoising or generative models where the goal is to generate physically realistic 3D structures that respect rotational or reflection symmetries.

### A.2. Metrics description

This subsection provides a detailed explanation of the metrics used to evaluate the performance of similarity search methods and ranking strategies in our study.

#### A.2.1. Hit rate reduction

Hit rate reduction (%) measures the proportion of the search space eliminated by prioritizing approved drugs within higher ranks in the list of compounds. It is calculated as:

$$\left(1-\frac{\text{Rank}\;\text{of}\;\text{First}\;\text{Approved}\;\text{Drug}}{\text{Total}\;\text{Number}\;\text{of}\;\text{Compounds}}\right)\times 100$$

The reported value represents the average hit rate reduction across all drug–target pairs in the dataset. Higher values indicate better prioritization of approved drugs, effectively narrowing down the search space and improving computational efficiency.

Run-time (s). This metric represents the average time required for a similarity search method to compute all SSs for a single protein target across the entire dataset of molecules. Specifically, it measures the total time taken for embedding generation and pairwise similarity computations for one protein target. The reported value is the mean run-time across all protein targets in the dataset.

Docking time (s). Docking time measures the average time needed to perform docking simulations to identify potential drugs. For each protein target, this metric calculates the average docking time required to process molecules from the top of the ranked list down to the first approved drug (first hit). This value is then averaged across all protein targets in the dataset. The purpose of this metric is to evaluate how effectively a similarity search method prioritizes suitable molecules for docking. Since docking simulations for unsuitable molecules often take significantly longer, this metric highlights the ability of our method to rank potential drugs higher, thereby reducing computational costs and accelerating the discovery process.

These metrics collectively provide a comprehensive evaluation framework, balancing accuracy (hit rate reduction) and computational efficiency (run-time and docking time) to assess the suitability of various methods in the drug repurposing pipeline.

### A.3. Experimental details

All experiments in this study were conducted using NVIDIA A100 GPUs, each with 40 GB of memory, providing the computational power required to handle large-scale datasets and perform deep learning-based similarity search tasks efficiently. The combination of high memory capacity and processing throughput enabled seamless model execution and consistent performance benchmarking across various configurations. Notably, QVina-W docking was executed using an AMD EPYC 7713 64-Core Processor with 128 CPUs, leveraging its high parallelism to maximize docking efficiency. To ensure standardized and reproducible performance assessments, all time measurements, including those for docking and similarity search, were obtained under this setup. To further support reproducibility, we provide the key software tools and dataset versions used in this study, including the DrugBank dataset (version 5.1.12, released March 14, 2024), AutoDock Vina (version 1.2.5), and FPocket (version 3.1.3). A complete list of package dependencies and environment specifications is available in our public repository: DrugPipe.

### A.4. Additional results

This subsection aims to provide further insights into the performance of various similarity search methods tested on the DrugBank DTI dataset. As illustrated in Fig. A1, the distribution of protein targets based on the ranking position of their first approved drug across different methods reveals distinct patterns of effectiveness. Morgan fingerprints demonstrate a slightly better focus on higher-ranked positions compared to GNNs and Equiformer, aligning with their strong performance in prioritizing approved drugs. EGNNs display the sharpest concentration of approved drugs in top-ranked positions, confirming their superior ability to identify relevant compounds. Meanwhile, GATs exhibit a balanced distribution, further highlighting their consistency across ranking tasks.

In addition to ranking positions, we evaluated the ADMET (Absorption, Distribution, Metabolism, Excretion, and Toxicity) properties [58] of drugs for the ranked lists generated in two case studies: HIV and COVID-19. ADMET properties are critical for assessing drug potential, as they provide insights into a compound’s pharmacokinetic and safety profile, which are essential for evaluating its suitability as a therapeutic candidate. Due to the size of the resulting tables, detailed results are available in our public repository DrugPipe, where readers can explore the complete data.
